# Chromosome Evolution and Genome Miniaturization in Minifish

**DOI:** 10.1371/journal.pone.0037305

**Published:** 2012-05-16

**Authors:** Shaojun Liu, Tan Heok Hui, Sze Ley Tan, Yunhan Hong

**Affiliations:** 1 Key Laboratory of Protein Chemistry and Developmental Biology of State Education Ministry of China, College of Life Sciences, Hunan Normal University, Changsha, China; 2 Department of Biological Sciences, National University of Singapore, Singapore, Singapore; University of California Los Angeles, United States of America

## Abstract

**Background:**

*Paedocypris* is a newly established genus of fish in Southeast Asia. *Paedocypris* is characterized by several unique features, including a tiny adult size (thus named miniature fish or minifish), fragmentary habitats of acidic peat blackwater swamps, an unusual reproduction mode and truncated development. These peculiarities lend themselves excellent for studying chromosome evolution and rapid speciation in vertebrates but also make them highly controversial for the phylogenetic position.

**Methodology and Principal Findings:**

We have established an organ procedure to prepare chromosome spreads from tiny organs of minifish and performed a cytogenetic study on two species of the genus *Paedocypris*, namely *P. carbunculus* (Pc) and *P. sp. “Singkep”* (Ps). We found 30 and 34 chromosomes in diploid cells of Pc and Ps, respectively, which are unusual in teleost fishes. The diploid metaphase has 5 pairs of metacentrics and 7 pairs of subtelocentrics in Pc compared to 3 pairs of metacentrics and 11 pairs of subtelocentrics in Ps, whereas the haploid metaphase contains 5 metacentrics and 7 subtelocentrics in Pc compared to 3 metacentrics and 11 subtelocentrics Ps. Chromosome behavior in first meiosis revealed the presence of a chromosomal ring consisting of 2 metacentrics in Pc, suggesting that centric fusion rather than fission was responsible for the karyotypic evolution from Ps to Pc. Flow cytometry revealed that Pc had a 45% nuclear staining intensity relative to medaka whose genome is 700 Mb in size and contains 0.81 pg DNA. The Pc genome should have 315 Mb in length and 0.36 pg of DNA, which represent one of the smallest values in vertebrates, suggesting genome miniaturization in this organism.

**Conclusions:**

Our data demonstrate that gross chromosome rearrangements and genome miniaturization have accompanied the evolution of *Paedocypris* fishes. Our data also place *Paedocypris* outside currently described taxa of the Cypriniformes.

## Introduction

The genus *Paedocypris* is a newly established group of miniature fishes with the discovery of two species, namely *P. micromegethes* and *P. progenetica* [1]. A third species, *P. carbunculus*, has recently been described [1]. There are additional miniature populations/species of this genus, which remain to be described, from other areas of Southeast Asia, such as Pulau Singkep, Pontianak, Pulau Banka and Kalimantan Tengah [1], [2]. These fishes inhabit tannin-stained, highly acidic (ca. pH 3∼5) peat swamp and flooded forests or blackwater swamps of Southeast Asia. They represent the smallest mature adult vertebrate species, as one of the first two described species matures at only 7.9 mm standard length (*P. progenetica*) [3]. Besides a tiny body size and a highly acidic habitat, *Paedocypris* features an unusual reproductive mode and truncated development, making this fish group excellent model for studying the mechanism and evolution of miniaturization, acidic adaptation, reproductive behavior and body plan in vertebrates.

Although initially placed in the family Cyprinidae of the order Cypriniformes on the basis of morphological data [3], *Paedocypris* is a subject of considerable controversy on the phylogenetic position. *Paedocypris* species can easily be distinguished from all other cypriniforms and even all other teleosts by uniquely modified pelvic fins in males: The first pelvic fin ray is greatly enlarged to support the keratinized pads of the skin, forming a structure called “flange and hook” [3]. Furthermore, *Paedocypris* species possess a highly modified anatomy consisting of developmentally truncated characters and highly derived autapomorphic features. The unusual anatomical characters make it difficult to assign *Paedocypris* to any subfamilies of Cyprinidae with confidence in morphology [1], [3], [4]. A molecular study by using sequence data of the mitochondrial cytochrome b gene has suggested that *Paedocypris* belongs to the Rasborinae clade and its closest relative is *Sundadanio*, another cyprinid also from Southeast Asia [2]. This placement was supported by a detailed osteological analysis of unnamed *Paedocypris* species from Banka [4]. *Sundadanio* and *Paedocypris* are both restricted to the highly fragmented peat swamp habitats in Southeast Asian. It deserves to note that *Paedocypris* species exhibit extensive divergence, as the four *Paedocypris* populations from different peat swamps exhibit considerable genetic differences [2]. A more recent molecular analysis by comparing DNA sequences of six nuclear genes has, however, proposed that *Paedocypris* stands outside the Cyprinidae and other families of the Cypriniformes [5]. Therefore, the phylogenetic position of *Paedocypris* has remained unresolved.

In evolutionary biology, there is a widely accepted hypothesis that small populations favor rapid speciation and chromosomal evolution [6]. This hypothesis has been supported in diverse animal taxa including mammals, where population structure is strongly correlated with speciation rate and chromosomal evolution: Taxa with high karyotypic diversity and rapid speciation rates may generally have small populations, whereas large populations may be associated with constant karyotypes and slow speciation rates [7]. Cytogenetic analyses of chromosomes and cellular DNA contents have long been used for taxonomy, phylogeny and evolution in diverse fish taxa [8], [9], [10]. Accumulated data show that fishes fall into two major groups. Conservative groups have constant chromosome numbers and are characterized by highly vagile species and a large population, whereas variable groups exhibit wide variability in chromosome number and are often associated with a small population [8], [9], [10], [11]. Featuring a tiny body size, fragmentary habitats and small populations [3], *Paedocypris* offers an excellent model system to study chromosome variation and organism evolution. However, chromosome analysis has so far been absent in minifish species, probably because of the elusiveness to obtain live specimen for laboratory experimentation and more importantly, the difficulty to obtain a sufficient amount of appropriate tissues and cell samples for chromosome preparation.

In this study, we developed procedures and analyzed chromosomes and DNA content in *Paedocypris*. We show that two *Paedocypris* species are easily distinguishable in chromosome number from each other and from other teleosts. Strikingly, we reveal that the *Paedocypris* genome represents one of the smallest known vertebrate genomes. These cytogenetic data suggest that chromosome rearrangement and genome miniaturization have accompanied the evolution of minifishes in the genus *Paedocypris*. Collectively, these results suggest the placement of *Paedocypris* outside all the described families of Cypriniformes.

## Results

### Chromosome preparation procedure

**Figure 1:**
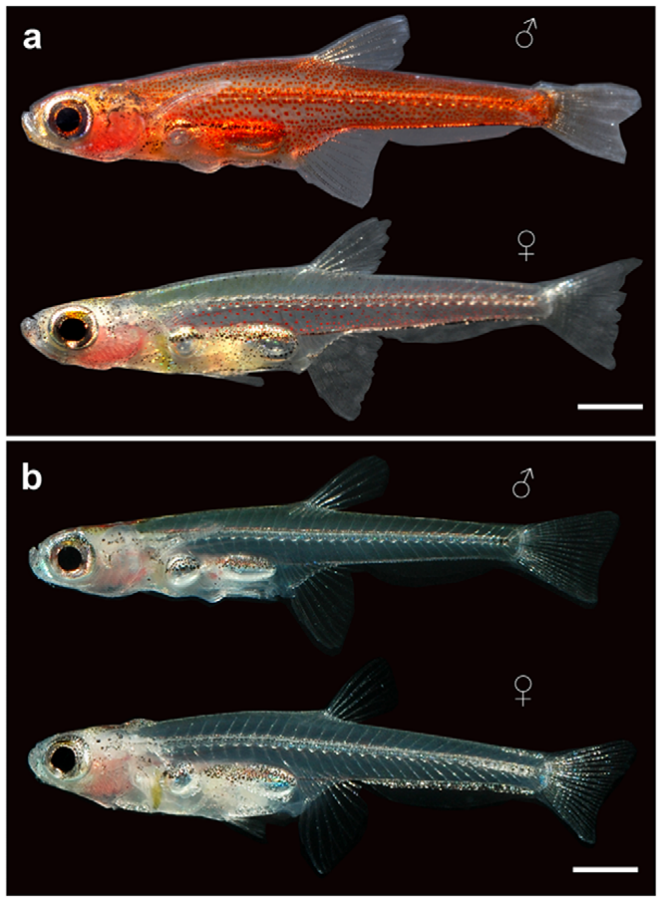
*Paedocypris* and cytogenetic analyses. (A) Live *Paedocypris carbunculus* (Pc) male (top) and female (bottom), preserved in 95% ethanol. Arrow depicts the fin fold resembling that appearing at larval stages. (B) Live *Paedocypris sp. “Singkep”* (Ps) male (top) and female (bottom), not preserved. Scale bar, 1 mm.

The two species we studied were *P. carbunculus* (Pc) collected from the Central Kalimantan in Indonesia and *Paedocypris sp. “Singkep”* (Ps) island, the latter being a species to be described. They have a standard length of ∼10 mm and certain larval characters such as the fin fold (Figure 1).

**Figure 2:**
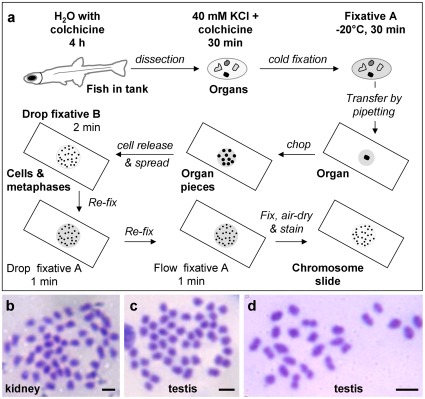
Organ procedure of chromosome preparation. (A) Flowchart illustrating major steps of the organ procedure. This procedure does not require centrifugation and allows for chromosome preparation from tiny organs. All steps are done at room temperature (23∼31°C) unless indicated. For details see Materials and Methods. (B–D) Metaphases by using the organ procedure in medaka as the test organism, showing diploid metaphase of 48 chromosomes from the kidney (B) and testis (C) as well as metaphase of 24 chromosomes from the testis (D). Scale bars, 5 µm.

A tiny size prevents chromosome preparation from the tissues and organs of minifish by using the existing procedures. We developed two procedures for chromosome preparation from entire organs or dissociated single cells. The organ procedure is rapid and simple (Figure 2A), which was modified from the previous procedure for rapid chromosome preparation from a single fish embryo [12]. When tested in medaka, this procedure produced metaphases from the kidney and testis (Figure 2B–D), as well as other organs such as the fin, spleen and ovary (data not shown). In some of these metaphases, 48 chromosomes were clearly countable in mitotic cells (Figure 2B and C), and 24 chromosomes were seen in meiotic cells (Figure 2D), respectively, thus validating the usefulness of the procedure. Medaka has been reported to have 48 chromosomes per diploid cell and 24 chromosomes per haploid cell [13], [14], [15]. This procedure operates on entire organs and produces a high yield of metaphases. The cell procedure produced metaphases also from multiple organs. This procedure preserved chromosome morphology at the expense of a lower metaphase yield, obviously due to cell loss during repeated centrifugation and cell resuspension. Both procedures were successful in *Paedocypris*. We found that the adult testis and ovary were the best organs for chromosome preparation in *Paedocypris*.

### Distinct karyotypes in two Paedocypris species

**Figure 3:**
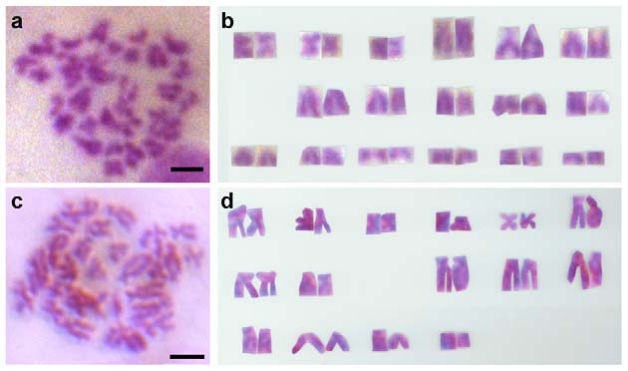
Diploid metaphases and karyotypes. (A and B) Ps mitotic metaphase from the ovary (A) and karyotype (B). (C and D) Pc mitotic metaphase from the testis (C) and karyotype (D). Scale bars, 5 µm.

We then determined the chromosome number in the two species. In Ps, 10 (53%) out of 19 diploid metaphases from the adult ovary were found to contain 34 chromosomes, and the remained had 29∼33 chromosomes, producing 34 for the modal diploid chromosome number for this species (Figure 3A). When cut from printed metaphases and arranged in pair to present the karyotype, individual chromosomes fall into three groups consisting of metacentrics (m), submetacentrics (sm) and subtelocentrics (st). Telocentrics (t) are absent. This karyotype analysis revealed the presence of 6 m, 6 sm and 22 st in the diploid cell of this species (Figure 3B), and a number of 46 for the fundamental number of chromosome arms (NF; m and sm are bi-armed, while st and t are uni-armed), thus producing a chromosome formula as 2n = 34 = 6m+6sm+22a. In Pc, 17 (61%) out of 28 diploid cells counted harbored 30 chromosomes, and the remainder had 22∼29 chromosomes. Therefore, Pc possesses a modal diploid chromosome number of 30 (Figure 3C). A karyotype analysis revealed a chromosome formula as 2n = 30 = 10m+6sm+14a, NF = 46 (Figure 3D). Therefore, Pc and Ps are clearly distinguishable in chromosome number and karyotype. Importantly, a difference in diploid number by 4 chromosomes accompanies a difference in number of metacentrics by 4 without a change in NF.

**Figure 4:**
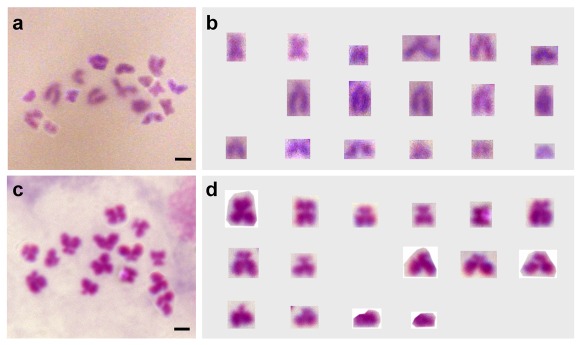
Haploid metaphases and karyotypes from the adult testis. (A and B) Ps meiosis-II metaphase (A) and karyotype (B). (C and D) Pc meiosis-II metaphase (C) and karyotype (D). Scale bars, 5 µm.

**Figure 5:**
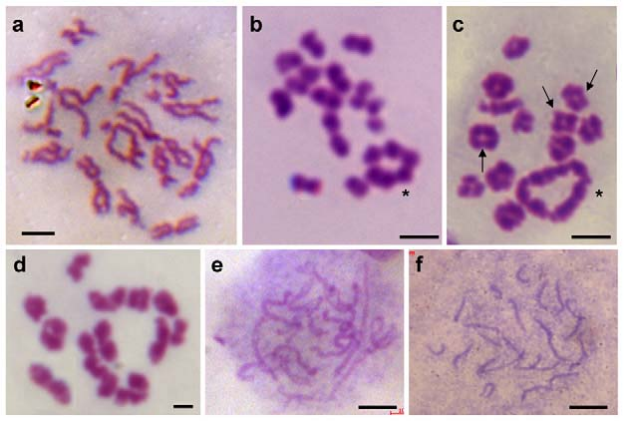
Chromosome morphology and behavior during meiosis. (A) Early meiosis-II metaphase from the Pc testis, highlighting the absence of telocentrics. (B and C) Meiosis-I metaphase from the Pc testis, highlighting the presence of a ring-like multivalent consisting of two metacentrics (asterisks). (D) Meiosis-I metaphase from the Ps testis. (E) Meiosis-I prophase from the Ps ovary. (F) Meiosis-I prophase from the medaka ovary. No multivalent is seen in Ps and medaka. Individual metacentrics are indicated by arrows. Scale bars, 5 µm.

The chromosome numbers and karyotypes were further examined in meiosis-II metaphases, which are easier for observation and precise counting because of fewer chromosomes per cell (Figure 4A and C). This revealed chromosome formulae as n = 17 = 3m+3sm+11st for Ps and n = 15 = 5m+3sm+7st for Pc (Figure 4B and D), respectively. Due to reduction division in meiosis I, meiosis-II metaphases are expected to contain one single set of chromosomes rather than two sets in mitotic metaphases. The haploid numbers and karyotypes thus represent a confirmation of the results in diploid cells. In addition, a closer inspection in early meiosis-II metaphases revealed the absence of telocentrics chromosomes in Pc (Figure 5A).

### Chromosome rearrangement in *Paedocypris*

Both the *Paedocypris* species analyzed here are distinct in chromosome and karyotype among teleosts examined so far. Specifically, they have the same number of NF but different numbers of total chromosomes and metacentrics: A reduction in the chromosome number by 4 accompanies 4 more metacentrics and 8 fewer telocentrics. These features are strongly suggestive of centric fission or fusion events in the karyotypic evolution. In order to investigate whether centric fission or fusion occurred in the evolution of *Paedocypris*, we examined the chromosome behavior during meiosis of both Pc and Ps. It has been reported in the amphibian species *Physalaemus petersi* that chromosomes resulting from recent centric fusion or fission events exhibit multivalent configurations, leading to the formation of rings or chains at meiosis I [16]. We observed a circular multivalent comprising the two largest metacentrics in Pc meiosis-I prophases (Figure 5B and C), the number of extra metacentrics in Pc compared to Ps. Such a multivalent was absent in male (Figure 5D) and female meiosis-I prophases (Figure 5E) of Ps as well as in male (Figure 2C) and female meiosis (Figure 5F) of medaka, a species that has been thought of retaining the basal chromosome number of the taxon to which it belongs [17]. The two metacentrics forming the ring-like multivalent in Pc meiosis are very possibly the products of recent centric fusion events from 4 subtelocentrics. The 2 centric fusion events might have generated 2 pairs of new metacentrics from 4 pairs of subtelocentrics, and reduced the diploid chromosome number by 4 from 34 in Ps to 30 in Pc. Therefore, centric fusion has been the major cause for the karyotypic evolution between the two *Paedocypris* species. In this regard, Ps appears to be more primitive, whereas Pc is more advanced.

### Genome miniaturization in Paedocypris

**Figure 6:**
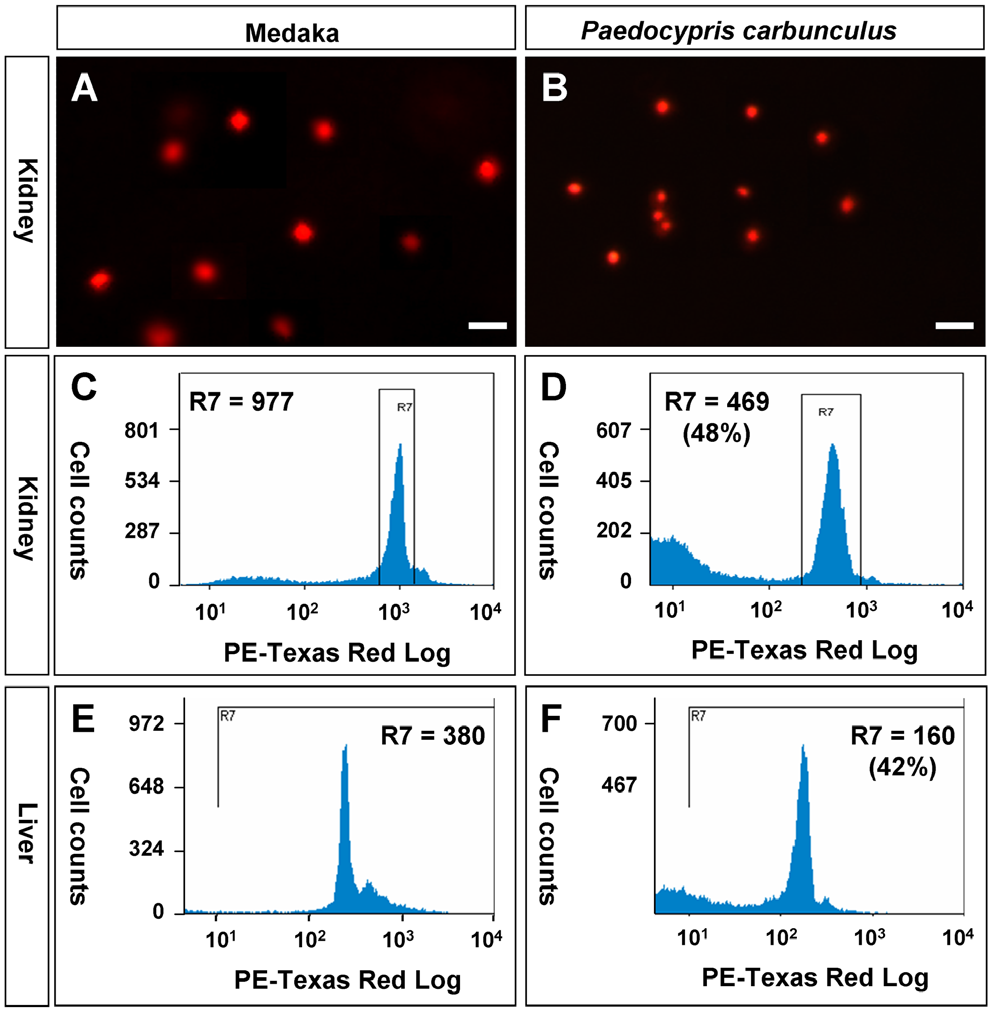
Nuclear size and DNA content estimation. (A and B) Kidney cells after nuclear staining with PI (red). (C and D) Flow cytometry profile of kidney cells. (E and F) Flow cytometry profile of liver cells. The means of relative staining intensity in the framed area (R7) are given. Percentage values (D and F) are the relative values of Pc compared to medaka, namely (C) versus (D), and (E) versus (F).

We wanted to determine the genome size in Pc as a representative of *Paedocypris*. Single cells were pooled from the liver of ten adult fish and stained for nuclei with the fluorescent dye propidium iodide (PI) for microscopy and flow cytometry. Similarly, medaka kidney cells were prepared for comparison. We observed that nuclei from medaka (Figure 6A) were apparently larger (∼2 fold) than nuclei from Pc (Figure 6B). Under the current conditions of flow cytometry analysis, the medaka kidney cells produced a relative signal intensity of 977 (Figure 6C), whereas the Pc kidney cells gave rise to 469 (Figure 6D), which corresponds to 48% of the medaka value. When liver cells were similarly used, the values read 380 and 160 for medaka and Pc (Figure 6E and F), respective, producing a relative value of 42% for Pc. On average, the relative nuclear staining was 45% in Pc compared to medaka.

The medaka genome has been reported to contains ∼700 Mb [18], thus a calculated DNA content of 0.81 pg (1 pg = 921 Mb; http://en.wikipedia.org/wiki/C-value). This calculated DNA content is in accordance with 0.85 pg determined by cytometry [17]. Therefore, the Pc genome was estimated to be 315 Mb in length (700 Mb×45%) and to contain 0.36 pg of DNA. The smallest vertebrate genome has recently been described in *Sphoeroides spengleri*, a species of the Tetraodontifomes, which measures 0.34 pg of DNA [19]. Consequently, together with *Sphoeroides spengleri*, the minifish Pc features the smallest known vertebrate genome. Since the DNA content is ≥1 pg in most fish species, genome miniaturization must have accompanied *Paedocypris* evolution.

## Discussion

In this study, we have determined the karyotype and estimated the genome size in *Paedocypris*. We have revealed a salient difference in chromosome number and karyotype between the two *Paedocypris* species, Pc and Ps, demonstrating the usefulness of chromosome analysis for species distinction in this taxon. Interestingly, the 30 chromosomes of Pc contain 5 pairs of metacentrics and 7 pairs of subtelocentrics, compared to the 34 chromosomes of Ps that contains only 3 pairs of metacentrics and up to 11 pairs of subtelocentrics, with NF being unchanged in both species. A variation in chromosome number without a change in NF is characteristic of the consequences from centric fission or fusion. Furthermore, we have observed the presence of a ring-like multivalent comprising 2 metacentrics in meiosis I of Pc, which is absent in Ps. This strongly suggests that the major mechanism underlying in the karyotypic evolution of *Paedocypris* species is centric fusion rather than fission. The ability of the 2 extra metacentrics in Pc to form a multivalent also implies their recent origin by centric fusion, because such chromosomes resulting from recent centric fusion or fission events have been reported to be able to take multivalent configurations and form rings or chains at meiosis I [16]. It holds equally true that the ring chromosome would the consequence of centric fission. Hence, Pc appears to be more advanced than Ps and should represent an evolving species or vice versa. At present, it is impossible to evaluate to what extent the chromosome numbers and karyotypes we have obtained in the 2 minifish species may be representative of the genus *Paedocypris*. Future work is needed to determine whether centric fusion/fission has been limited to Pc and Ps or operated widely also in other *Paedocypris* species. In this regard, the organ procedure established in this study will provide a tool for chromosome studies in minifish species. This procedure operates on entire organs without a need for centrifugation and cold wet slides (which are required for chromosome preparation by air-drying method), and should be applicable for field work studies.

The cytometry analysis has shown that Pc has a 0.36 pg haploid DNA content and thus an estimated genome size of 315 Mb. Since many teleost species examined so far have ≥1 pg DNA, genome miniaturization by extensive DNA loss must have happened in *Paedocypris* after its split from the lineage leading to other taxa. It deserves to note that the small genome of Paedocypsis brings novelties because it occurs in a group of the Tetraodontiformes [19]. Understanding of the mechanisms underlying genome miniaturization in both *Paedocypsis* and Tetraodontiformes will shed important lights on the evolution of a minimal genome in vertebrates.

As *Paedocypris* has several unique characters including body miniaturization, adaptation to unusually acidic habitats, special reproductive mode and developmental truncation [1], [3], [4], [5], [20], it will be interesting to determine whether genome miniaturization is associated with the evolution of one or all of the unique characters. A tiny adult size and more importantly, truncated development may account for genome miniaturization in *Paedocypsis*, for genes required for continuous growth and development of adult characters such as the head bone and fins might be permissive for loss without considerably affecting the fitness. If this would hold true, *Paedocypsis* would represent a first model organism to define the minimal number of genes essential for development and fitness.

**Figure 7:**
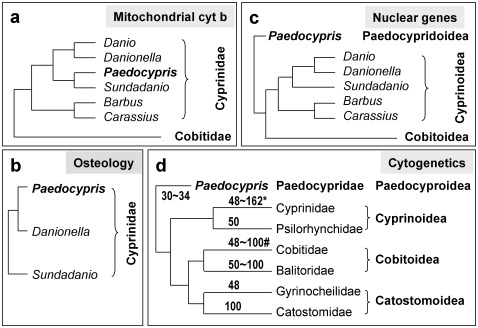
Phylogenetic position of *Paedocypris* relative to Cyprinidae. (A) Positioning of *Paedocypris* within Cyprinidae on the basis of mitochondrial cytochrome b sequence data [2]. (B) Positioning of *Paedocypris* within Cyprinidae on the basis of morphological data [4]. (C) Positioning of *Paedocypris* outside Cyprinidae on the basis of 6 nuclear gene sequence data [5]. (D) Cytogenetic positioning of *Paedocypris* outside Cyprinidae on the basis of chromosome data (this study). Notably, Paedocyroidea is sister to all the three classical superfamilies. Diploid chromosome numbers are given for each taxon. *Exceptions are present in the subfamily Acheilognathinae, in which 6 of 10 species examined have 2n = 42∼46 chromosomes. #Exception is present in 2 species, which have 2n = 40 and 44 chromosomes.

The results obtained have an important implication for the phylogenetic relationship between Paedocypris and other taxa. Paedocypris has been placed in the family Cyprinidae on the basis of mitochondrial cytochrome b sequence data (Figure 7A) [2] and osteological data (Figure 7B) [4]. The genome size exhibits apparent variations in North American minnows (Cyprinidae) [21]. For example, the mean genome size of *Iberochondrostoma almacai* (Teleostei, Cyprinidae) is statistically significantly smaller than that of its sister species *I. lusitanicum*, 2.61 and 2.93 pg, respectively [21], [22]. A recent investigation by using nuclear genes has proposed that *Paedocypris* is a monophyletic group sister to all taxa of the Cypriniformes (Figure 7C), not members of the family Cyprinidae [5]. Our cytogenetic data in combination with reported morphological data allow for re-consideration of the phylogenic position of *Paedocypris*. *Paedocypris* is morphologically placed in the order Cypriniformes [3], [4]. Cytogenetically, *Paedocypris* cannot be placed in any cypriniform taxa at subfamily, family and superfamily levels (Figure 7D). Currently, six families are placed in three superfamilies, namely Cobitoidea, Catostomoidea and Cyprinoidea, with the Cyprinoidea comprising Cyprinidae and Psilorhynchidae [23]. Extensive cytogenetic studies in Cypriniformes have revealed chromosome numbers in more than 200 species belonging to all the six families, most of them have ≥48 chromosomes [10], [11], [24]. With 30∼34 chromosomes in diploid cells, *Paedocypris* clearly distinguishes itself from the three described superfamilies. If *Paedocypris* indeed belongs to the Cypriniformes, it forms a new superfamily, Paedocyproidea (Figure 7D). This placement is similar to that inferred from nuclear gene sequences (Figure 7C). However, cytogenetic data cannot determine whether *Paedocypris* stands within or outside Cypriniformes. Future cytogenetic studies in more miniature species, in particular those of genera *Paedocypris*, *Sundadanio* and *Danionella*, will provide instructive information.

In summary, fishes of *Paedocypris* have the smallest known vertebrate body size and the smallest known vertebrate genome. Amazingly, our work has established *Paedocypris* as a first model for the involvement of both chromosome rearrangements and genome miniaturization in vertebrate evolution. We show that *Paedocypris* is not a taxon within the family Cyprinidae but instead forms a distinct taxon whose phylogenetic position remains to be elaborated in the future.

## Materials and Methods

### Fish

Work with fish followed the Guidelines on the Care and Use of Animals for Scientific Purposes of the National Advisory Committee for Laboratory Animal Research in Singapore. Work with fish in this study was specifically approved by the National Advisory Committee for Laboratory Animal Research in Singapore. Work with medaka was specifically approved under permit number 27/09. Work with minifish was specifically approved under permit number 065/06. No issue on ethics was concerned in this study. Medaka was maintained as described [14], [15]. *Paedocypris* samples were supplied by a local dealer. *P. carbunculus* (Pc) [1] was collected from the Central Kalimantan in Indonesia, and a species tentatively called *Paedocypris sp. “Singkep”* (Ps) was from the Sumatran Singkep Island. The fish were maintained at ambient temperatures (23∼31°C) in glass tanks containing 5∼20 L of acidic water (peat filtered water; ∼pH 4.5) under an artificial photoperiod of 14-h/10-h light/darkness at 26°C. Fish were fed twice a day with live embryos of brine shrimps (*Artemia nauplii*) plus commercial dry food (RNS225).

### Flow cytometry

The kidney and liver were dissected from 10 adults of *Pc* and manually dissociated into single cells by tearing with a pair of tweezers followed by gentle pipetting in a 2-cm dish containing 1 ml of phosphate buffered saline (PBS). The sample was collected into a 1.5-ml Eppendorf tube and allowed to stand for 5 min. The single cell suspension was transferred into a new 1.5-ml tube and spun for 2 min at 6000 rpm in a benchtop centrifuge (Eppendorf). PBS was removed, and the cell pellet was resuspended in 300 µl of PBS. The cells were chilled on ice and fixed by adding 700 µl of cold 100% ethanol (4°C). For flow cytometry, the cells were incubated at 37°C for 15 min in PBS containing RNase A (200 µg/ml) and propidium iodide (20 µg/ml) for DNA staining, and analyzed with a Coulter Elite ESP flow cytometer equipped with a WinMDIv2.8 software (Beckman Coulter) as described [13], [15]. The medaka kidney cells were similarly treated as a reference for comparison.

### Chromosome preparation

An organ procedure was established for preparation of metaphases from whole organs of minifish, which was modified from a procedure for rapid preparation of chromosomes from single fish embryos [12]. Adult fish were reared at ambient temperatures in acidic water containing 50 µg/ml colchicine in darkness for 4 h. Entire organs (gill, kidney and gonad) were manually dissected under a stereomicroscope by using fine tweezers and a pair of scissors. The organs were hypotonically treated for 30 min at room temperature (25°C) in 2-cm Petri dishes with 5 ml of 40 mM KCl containing colchicine (final 50 µg/ml). After replacement of KCl with 5 ml of cold (−20°C) fixative A (Carnoy's fixation by freshly mixing methanol and acetic acid at a 3∶1 ratio in volume), the dishes were put into a freezer (−20°C) and treated for 15∼30 min. Shortly before the completion of this treatment, fixative B was prepared by mixing cold methanol (−20°C) and 75% acetic acid at a 1∶1 volume ratio. An organ was transferred by pipetting together with ∼50 µl fixative A onto a dry slide and immediately chopped quickly with tweezers to separate the organ into small aggregates and single cells. During chopping, fixative A with cells was kept within a round filed of ∼1-cm in diameter to reduce cell dilution and loss. Fixative B (∼100 µl) was dropped onto the area of fixative A on the slide. During a 2-min period in fixative B, tissue pieces rotated quickly, and single cells were released and spread. Freshly prepared fixative A (100 µl) was dropped onto cells. After treatment for 1 min, the slide was placed at a ∼45° angle, and fixative A was dropped to flow over the cells. The slide was finally immersed in fixative A for 15 min followed by air drying and staining with 5% Giemsa in 5 mM phosphate buffer (pH 6.8) for 20 min. After washing in distilled water, the slide was used for microscopic observation.

### Karyotype analysis

Metaphases were counted for each species. Metaphases were chosen on the basis of cellular intactness and chromosome morphology, and chromosomes on printed metaphases were cut and grouped according to the ratio between the long and short arm [25]: Metacentrics (ratio 1∼1.7) and submetacentrics (ratio 1.8∼3.0) were considered as bi-armed chromosomes, subtelocentrics (ratio 3.1∼7.0) and telocentrics (ratio >7) were considered as uni-armed chromosomes. Bi-armed and uni-armed chromosomes were separated with a blank as described [11], [26], [27].

### Microscopy

Fish were observed and photographed on Leica MZFIII stereo microscope with a Nikon coolpix4500 digital camera, and cells and chromosomes were observed and photographed on a Zeiss Axiovert upright microscope with a Zeiss AxioCam M5Rc digital camera as described [28], [29], [30].
